# AI-based clinical decision support systems for antibiotic therapy in common adult infections: a systematic review from development to clinical implementation

**DOI:** 10.3389/fdgth.2026.1944745

**Published:** 2026-09-30

**Authors:** Miguel Hueda-Zavaleta, Rodrigo Flores-Palacios, Fabrizzio Bardales-Silva, Eric Cotacallapa-Pino, Ari Frenkel, Diana Minchon-Vizconde, Juan Carlos Gomez de la Torre Pretell

**Affiliations:** 1 Facultad de Ciencias de la Salud, Universidad Privada de Tacna, Tacna, Perú; 2 Hospital Daniel Alcides Carrión, Essalud, Tacna, Perú; 3 Facultad de Ciencias de la Salud, Universidad Nacional Jorge Basadre Grohmann, Tacna, Perú; 4 Arkstone Medical Solutions, New York, NY, United States; 5 Facultad de Medicina, Humana Universidad Ricardo Palma, Lima, Perú; 6 Laboratorio Clínico Roe, Lima, Perú

**Keywords:** antibiotic therapy, antimicrobial stewardship, artificial intelligence, bacteremia, clinical decision support system, implementation science, pneumonia, systematic review

## Abstract

**Background:**

Antimicrobial resistance is among the leading causes of global mortality, and artificial intelligence (AI)-based clinical decision support systems (CDSS) have been proposed to guide antibiotic prescribing. Several AI models for antibiotic decision-making have been developed and validated, but their actual deployment in clinical practice and effect on patient outcomes have not been systematically characterized.

**Objective:**

To identify, classify, and appraise studies evaluating AI-based CDSS for antibiotic therapy in hospitalized adults with common infections, stratified by level of clinical implementation.

**Methods:**

Search-strategy development began on June 7, 2026; definitive searches of PubMed/MEDLINE, Scopus, Web of Science Core Collection, and Embase were executed on June 28, 2026, after submission of the PROSPERO record. Four reviewers independently screened records and assessed reports. Studies were classified as deployed in clinical practice (Level 1), evaluated against physicians' decisions without deployment (Level 2), or tested through retrospective simulation (Level 3). Risk of bias was assessed with RoB 2, ROBINS-I, or PROBAST, as appropriate. Owing to clinical and methodological heterogeneity, findings were synthesized narratively, with an outcome-specific GRADE assessment.

**Results:**

Of 1,157 records identified, 10 studies met the inclusion criteria: three Level 1 (30%), four Level 2 (40%), and three Level 3 (30%). The three deployed studies evaluated pneumonia, predominantly pulmonary *Stenotrophomonas maltophilia* infection, and *Staphylococcus aureus* bacteremia and reported favorable signals for mortality, hospital processes, prescribing, or medication safety. Kanjilal et al., a published conference abstract, was included as a Level 3 retrospective evaluation of 29,508 adults with community-onset sepsis. Among the seven Level 2/3 studies assessed with PROBAST, three were classified as low, three as moderate under the prespecified descriptive adaptation, and one as high overall risk of bias. Certainty was low for mortality/survival and very low for the remaining clinically relevant outcomes.

**Conclusions:**

Three of 10 included studies reached clinical deployment, whereas seven remained non-deployed. Deployed systems showed favorable outcome and process signals, but the evidence was heterogeneous and of low to very-low certainty, depending on the outcome. Prospective multicenter evaluations of deployed systems are required before computational performance can be interpreted as clinical effectiveness.

**Systematic Review Registration:**

https://www.crd.york.ac.uk/PROSPERO/view/CRD420261435593, identifier CRD420261435593.

## 1 Introduction

Antimicrobial resistance (AMR) has become one of the most pressing threats to global public health. The Global Burden of Disease Study estimated that bacterial AMR was directly responsible for 1.27 million deaths and associated with 4.95 million deaths worldwide in 2019, a figure comparable to major causes such as HIV/AIDS and malaria combined (1). In hospital settings, bloodstream infections, pneumonia, and urinary tract infections account for the largest proportion of antibiotic use and are also the clinical scenarios most affected by inappropriate prescribing and rising rates of resistance (2). Each course of unnecessary or inadequate therapy simultaneously jeopardizes the individual patient and accelerates the selection of resistant organisms at the population level, making the optimization of antibiotic decisions a dual clinical and public-health priority.

Appropriate empiric antibiotic therapy depends on the timely interpretation of microbiological data, local resistance patterns, and patient-specific factors. Conventional culture and susceptibility testing, however, requires 48–72 h, during which clinicians must rely on empiric choices that may prove inadequate. Delays in the initial appropriate antibiotic therapy have been associated with a 20% increase in mortality among patients with severe Gram-negative bacterial infections, regardless of resistance phenotype (3). This has motivated the need to develop clinical decision support systems (CDSS) that assist clinicians in the appropriate and timely selection of antibiotics (4).

Artificial intelligence (AI) and machine learning (ML) have attracted substantial interest as potential engines for antibiotic CDSS. These systems can integrate electronic health record data, microbiological results, and institutional resistance patterns to generate patient-specific treatment recommendations in real time (5). Reported applications span resistance-prediction algorithms, empiric-therapy optimizers, and comprehensive platforms that simultaneously guide diagnosis, risk stratification, and antibiotic selection (6, 7), and increasingly extend to generative large language models proposed as conversational stewardship aids.

Several reviews have mapped these applications and their reported performance, and some have begun to organize the evidence along gradients of methodological maturity (8, 9). Some CDSS are still being incorporated into clinical practice, moving past their phase of retrospective development and validation. This distinction is decisive because the majority of AI models in healthcare are never deployed: a recurring pattern is that high-performing algorithms are validated on retrospective datasets, compared against historical decisions in simulation, and then proposed for a clinical integration that rarely materializes (10). For AI-based antibiotic CDSS specifically, the gap between development and deployment may carry direct consequences for patient outcomes, since the potential benefits of these systems can only be realized when they are used in real clinical workflows to guide treatment decisions for actual patients.

A bedside model introduces requirements that performance indicators on their own cannot capture interoperability between EHR information systems, external and temporal validation, dynamic strategies against resistance patterns that shift over time, formal delineation of medico-legal liability, empirical validation of its adoption within the clinical care workflow.

Evidence generated during the clinical implementation phase differs substantially from that obtained through prior computer simulations and conflating both scenarios carries the risk of overstating readiness for clinical use and safety for immediate incorporation into actual medical practice. A framework that explicitly stratifies studies by their level of clinical implementation is needed to characterize where the field actually stands.

Previous comparative evidence has suggested that machine-learning approaches may improve selected performance measures over conventional risk-scoring systems in antimicrobial stewardship, particularly sensitivity and negative predictive value; however, substantial heterogeneity, inconsistent findings across analytical approaches, and limited external validation have also been reported (11). These findings underscore that improved algorithmic performance alone does not establish clinical effectiveness and support the need to evaluate whether such systems progress to real-world implementation and improve patient-relevant outcomes. The objective of this systematic review was to identify, classify, and critically appraise studies evaluating AI-based CDSS for antibiotic therapy in hospitalized adults with common infections, specifically pneumonia, urinary tract infections, bacteremia/bloodstream infection (BSI), and sepsis. Hospital-acquired infections were considered only when they fell within these clinical syndromes. We stratified included studies by their level of clinical implementation—from retrospective simulation, through prospective clinical evaluation, to real-world deployment—to map the current state of evidence and the translational gap from algorithm to bedside, and to identify the priorities most likely to close it.

## 2 Methods

This systematic review was developed in accordance with the guidelines of the PRISMA (Preferred Reporting Items for Systematic Reviews and Meta-Analyses) statement 2020 (12). The review protocol was registered in PROSPERO [CRD420261435593].

Development of the search strategy began on June 7, 2026. The PROSPERO record was submitted late on June 27, immediately before midnight; the system therefore displays June 28, 2026, as the official submission and registration date. Definitive database searches were executed during the early hours of June 28, after submission. During peer review, the record was amended to correct the database list (CENTRAL was removed because it was never searched, and Web of Science Core Collection was added), clarify prespecified concepts, align procedural fields with the methods actually used, and document the post-registration addition of outcome-specific GRADE.

### 2.1 Eligibility criteria

All studies that met our PICO question were included: Detailed eligibility criteria applied during screening and full-text assessment are provided in Supplementary Material S3.

#### 2.1.1 Population

Hospitalized adults (18 years or older) with pneumonia, urinary tract infection (UTI), bacteremia/bloodstream infection (BSI), or sepsis. Hospital-acquired infections were considered only when they fell within these clinical syndromes. Studies focusing exclusively on pediatric populations, outpatient settings, or non-infectious conditions were excluded.

#### 2.1.2 Intervention

An AI-based clinical decision support system was defined as a computerized system using data-driven learning, probabilistic inference, or data-driven optimization to generate or modify individualized antibiotic-treatment recommendations. This included systems for empiric antibiotic selection, culture-guided therapy adjustment, resistance prediction informing treatment, and antibiotic optimization. Purely deterministic guideline engines, conventional risk scores without a data-driven component, and manual stewardship interventions without algorithmic decision support were excluded. For hybrid systems, the AI/data-driven component was recorded separately from any conventional guideline or rule-based component.

#### 2.1.3 Comparator

Standard clinical practice without CDSS, physician prescribing decisions (prospective or retrospective comparison), or pre-implementation periods in before-after designs.

#### 2.1.4 Outcomes

At least one of the following: mortality (in-hospital, 30-day, or 1-year), length of hospital stay, time to appropriate antibiotic therapy, antibiotic concordance or appropriateness, coverage rates, antibiotic spectrum (narrowing or de-escalation), adverse drug events, or antibiotic costs.

#### 2.1.5 Study design

Primary studies of any design including randomized controlled trials, quasi-experimental studies (stepped-wedge, before-after), cohort studies, cross-sectional evaluations, and retrospective model evaluations with comparison to clinical practice. Systematic reviews, narrative reviews, editorials, protocols, qualitative studies, case reports, and instrument-only validation studies without clinical comparison were excluded.

### 2.2 Information sources and search strategy

We systematically searched PubMed/MEDLINE, Scopus, Web of Science Core Collection, and Embase. Search-strategy development began on June 7, 2026, and the definitive database searches were executed during the early hours of June 28, 2026, after submission of the PROSPERO record; no date or language restrictions were applied. CENTRAL was not searched. The strategy combined terms for (1) artificial intelligence and machine learning, (2) antibiotic therapy, and (3) pneumonia, urinary tract infection, bacteremia/bloodstream infection, and sepsis. Complete executed strings are provided in Supplementary Material S1. Reference lists of included studies and relevant reviews were hand-searched; authors or experts were not contacted. PubMed/MEDLINE, Web of Science, and Scopus included hard-clinical-outcome terms, whereas Embase did not; this asymmetry was considered when interpreting retrieval completeness.

### 2.3 Selection process

Four reviewers independently screened titles and abstracts. Two reviewers used a systematic, keyword-based screening approach applied to all records retrieved from the four databases, while the two reviewers independently performed a manual screening of records identified from the same databases plus additional hand-searching. Discrepancies between reviewers were resolved by discussion and, when necessary, by consultation with a third reviewer. Studies passing title and abstract screening were retrieved in full text and independently assessed by both reviewers against the eligibility criteria. Reasons for exclusion at the full-text stage were documented. (Supplementary Material S2)

### 2.4 Data collection and data items

Data were extracted independently by one reviewer using a standardized extraction form and verified by the second reviewer. For all included studies, we extracted: first author, publication year, country, study design, sample size, clinical setting, infection type, population characteristics (age, sex), CDSS name and technical description (AI/ML or probabilistic/optimization method, data inputs, outputs, and any conventional guideline component), comparator, implementation level, calibration, threshold selection, external or temporal validation, clinical net benefit or decision-utility measures when reported, harms or false recommendations, and all other reported outcomes.

Included studies were classified into three implementation levels based on the degree of clinical deployment achieved. The two reviewers responsible for full-text assessment assigned the levels using the documented implementation characteristics; disagreements were resolved by discussion and, when necessary, consultation with a third reviewer. If a study contained more than one implementation phase, the highest level of clinical use actually documented determined the primary classification, while lower-level analyses were retained in the data extraction.

The three-level classification is a pragmatic evidence-maturity continuum rather than a formal Technology Readiness Level instrument, although it conceptually aligns with clinical-AI readiness frameworks that distinguish model development, validation, prospective evaluation, and deployment (13). The implementation continuum from retrospective simulation through clinical evaluation to real-world deployment was prespecified in PROSPERO; the Level 1–3 labels were subsequently used to operationalize this prespecified continuum. Because PROBAST does not provide a standard ‘moderate' overall risk-of-bias category, we used this term descriptively when methodological concerns or insufficient reporting affected at least one domain but no domain was judged to be at high risk. Studies with at least one high-risk domain were classified as high overall risk, and applicability concerns were reported separately.

#### 2.4.1 Level 1 (clinical deployment)

The CDSS was deployed in actual clinical workflows and used by clinicians to guide antibiotic prescribing for real patients. Outcomes reflect the effect of CDSS use on clinical practice.

#### 2.4.2 Level 2 (clinical evaluation without deployment)

The CDSS generated recommendations that were compared with physicians' decisions in real clinical cases, but it was not integrated into the clinical workflow. Clinicians did not use the CDSS to guide their prescribing in real time.

#### 2.4.3 Level 3 (retrospective simulation)

The CDSS was applied retrospectively to historical patient data, and its recommendations were compared against what clinicians had actually prescribed. The comparison is entirely simulated, with no prospective evaluation of the CDSS against clinical practice.

### 2.5 Risk of bias assessment

Risk of bias was assessed independently by two reviewers using tools appropriate to each study design and implementation level. For Level 1 studies, we used the Cochrane Risk of Bias 2 (RoB 2) tool for randomized controlled trials (14) and the Risk of Bias in Non-randomized Studies of Interventions (ROBINS-I) tool for non-randomized intervention studies (15). For Level 2 and Level 3 studies, which evaluate prediction model performance rather than intervention effects, we used the Prediction model Risk Of Bias ASsessment Tool (PROBAST) (16). Disagreements between reviewers were resolved by consensus. Reporting bias was not assessed using funnel plots, as no outcome included ≥10 studies, in line with Cochrane Handbook recommendations.

### 2.6 Synthesis methods

We conducted a narrative synthesis of the included studies, stratified by implementation level. A quantitative meta-analysis was not performed for the following reasons: (a) the small number of included studies overall, and within each implementation level, precluded meaningful pooling; (b) the three Level 1 studies evaluated different CDSS platforms, targeted different infection types (pneumonia, S. maltophilia infections, and S. aureus bacteremia), employed different study designs (randomized trial, stepped-wedge trial, and before-after study), and reported heterogeneous outcome measures; and (c) Level 2 and Level 3 studies differed from Level 1 studies in their fundamental study question (prediction accuracy vs. clinical effectiveness), making cross-level pooling inappropriate.

The results of each study are presented descriptively, including original effect estimates and confidence intervals where available. At each level of implementation, we describe the clinical context and the characteristics of the clinical decision support system (CDSS) for comparability. We also synthesize the findings from all levels to characterize the timeline from model development to clinical implementation, ultimately identifying the implementation gap.

### 2.7 Certainty of evidence

We performed a structured, outcome-specific narrative GRADE assessment for the clinically deployed evidence. Two reviewers independently evaluated risk of bias, inconsistency, indirectness, imprecision, and publication bias, resolving disagreements by consensus. Meta-analysis is not required for GRADE; therefore, GRADE was applied to the narrative synthesis because statistical pooling was inappropriate. Formal outcome-specific GRADE was added during peer review and was not part of the original PROSPERO certainty-assessment plan. Ratings and domain justifications are reported in Supplementary Table S4.

## 3 Results

### 3.1 Study selection

The four database searches identified 1,157 records. After removal of 350 duplicates, 807 unique records were screened by title and abstract; 794 were excluded. Thirteen reports were sought and retrieved for eligibility assessment. Three reports were excluded: one secondary analysis of an already included cohort, one pediatric study, and one usability/acceptability study without comparative clinical outcomes. Kanjilal et al., previously listed among the exclusions, was reassessed and included because its retrospective comparison of model-generated and clinician antibiotic decisions met the Level 3 definition. Consequently, 10 studies were included in the narrative synthesis (Figure 1) (6, 7, 17–24).

**Figure 1:**
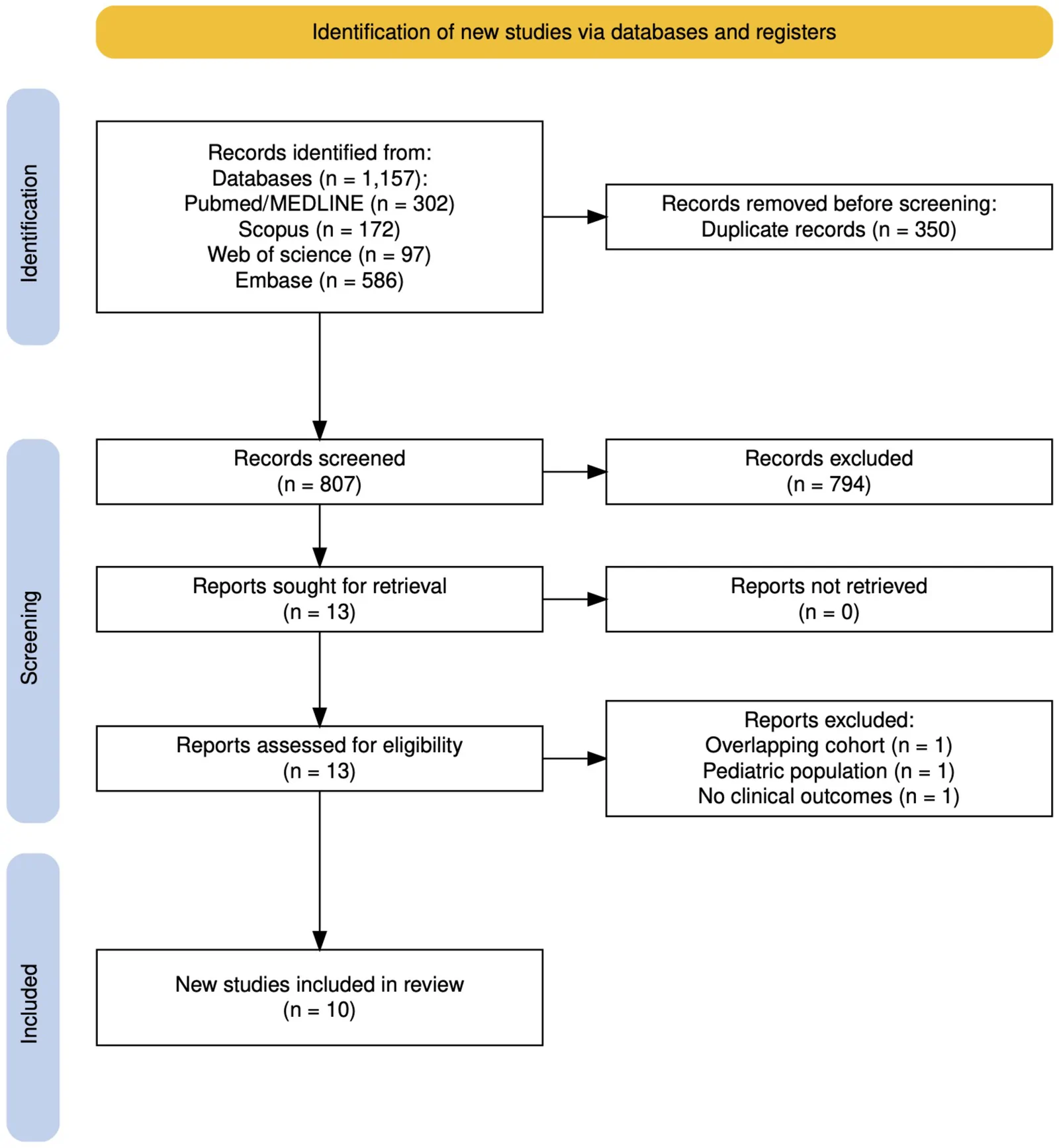
PRISMA 2020 flow diagram of the study selection process.

### 3.2 Study characteristics

The 10 included studies, detailed in Table 1, were published between 2015 and 2026; nine were published from 2022 onward. Four were conducted in the United States, two in Taiwan, two in Peru, one in South Korea, and one in China.

**Table 1:** Characteristics of included studies, stratified by implementation level.

Study	Country	Design	Setting	Infection (n)	Population	CDSS/AI method	Comparator	Impl. level	Primary outcomes	Key findings
Dean 2022 (7)	USA	Stepped-wedge cluster trial (NCT03358342)	16 community EDs, Intermountain	CAP (*n* = 6,848)	Median 67 yr; 48% F	ePNa: hybrid EHR-based CDSS; algorithmic risk stratification + conventional guideline-based antibiotic recommendations.	Pre-deployment care	Level 1: Deployed	30-d mortality; time to Abx; concordance; disposition	Mort aOR 0.62 (0.49–0.79, *P* < 0.001); concordance 83.5→90.2%; time to Abx −8.5 min; outpatient 29.2→46.9%
Lin 2025 (17)	Taiwan	RCT (ISRCTN16278872)	Tri-Service General Hospital	SM infections, 91% pulmonary (*n* = 1,600)	Mean 74.6 yr; 61.8% M	AI-CDSS: MALDI-TOF + ML (RF/LGBM/XGB); resistance 1d earlier	Standard practice (AST)	Level 1: Deployed	14-d mortality; prescribing confidence; Abx selection	Mort 11.5% vs. 15.1% (*P* = 0.03); confidence *P* < 0.001; Abx selection *P* < 0.001
Ho 2024 (18)	Taiwan	Before-after implementation	China Medical Univ Hospital	SA bacteremia MRSA/MSSA (*n* = 252)	Median 64.5 yr; 65.9% M	iAMS: AI platform in EHR; Abx recs + safety alerts	Pre-iAMS care	Level 1: Deployed	1-yr survival; LOS; discharge; medication incidents	MRSA survival 70.9 vs. 53.3% (*P* = 0.41); LOS < 14d *P* = 0.038; med incidents -97.3% (*P* = 0.013)
Gómez de la Torre 2026a (19)	Peru	Cross-sectional observational	Roe Lab + hospitals, Lima	Bacteremia (*n* = 366 eval; 35.5% E.coli)	94 physicians (35 ID, 59 non-ID)	OneChoice AI-CDSS: ML on FilmArray + MALDI-TOF/VITEK2	Physician decisions	Level 2: Clinical eval.	Concordance; *κ*; inappropriate prescribing	Concordance 96.1% (any), 74.6% (top); *κ*=0.70; ID κ=0.78 vs. non-ID κ=0.61
Gómez de la Torre 2025 (20)	Peru	Prospective cross-sectional	Roe Lab, Lima	Bacteremia (*n* = 117; 41% E.coli, 30% ESBL)	Median 67 yr; 58.1% M	OneChoice AI/ML CDSS: molecular vs. phenotypic recs	Mol-only vs. mol + phenotypic CDSS	Level 2: Clinical eval.	Concordance; turnaround time	Concordance 80.3%; κ=0.80; molecular 29 h earlier; E.coli 95%
Feng 2025^a^ (21)	China	Retrospective multicenter cohort	3 hematology centers	PA BSI onco-hematology (*n* = 924)	Median 41 yr; mort 10.6%	ML-CDSS: XGBoost/RF; resistance prediction + Abx algorithm	Physician decisions (retrospective)	Level 2: Clinical eval.	IET rate; carbapenem use; AUC	IET 11.9→4.0% (*P* = 0.01); 20.8→7.1% (*P* < 0.001); CRPA AUC 0.705–0.732
Lee 2023 (22)	South Korea	Retrospective single-center	Inha Univ Hospital ED	UTI in ED (*n* = 550; adults ≥18)	Adults; positive urine cx	GBDT: ciprofloxacin resistance + ESBL prediction; SHAP	Actual ED prescribers (retro)	Level 2: Clinical eval.	Ineffective Abx rate; AUC; features	Ineffective Abx -20%; narrowed to 5–20 predictors
Corbin 2022 (6)	USA	Retrospective multi-site	Stanford + ValleyCare + MGH/BWH	ED infections + UTI (*n* = 24,148)	Stanford 66 yr; Boston 52yr	ML antibiograms: XGBoost + linear programming	Clinician prescribing (simulation)	Level 3: Simulation	Coverage rate; spectrum narrowing	Stanford 85.9 vs. 84.3%; Boston 90.4 vs. 88.1% (*P* < 0.0001); 69% narrowed
Tsoukalas 2015 (23)	USA	Retrospective model + policy eval	UC Davis Medical Center	Sepsis (*n* = 1,492)	Hospitalized adults; EHR	POMDP: probabilistic Abx combination policies	Non-policy trajectories	Level 3: Simulation	Favorable transitions; mortality/LOS	Policy: 49% vs. 37% favorable (*P* = 1.3e-13); robust to variation
Kanjilal 2022^b^ (24)	USA	Retrospective ML/model evaluation	Mass General Brigham health system	Community-onset Adult Sepsis Event (*n* = 29,508)	Adults with community-onset sepsis	Logistic regression, RF, and XGBoost for pathogen/AMR prediction; dynamic thresholding to recommend the narrowest susceptible antibiotic	Clinicians' antibiotic decisions (retrospective)	Level 3: Simulation	Inappropriately narrow treatment; broad-spectrum use; pathogen/AMR performance	Inappropriately narrow: 7.3% model vs. 9.4% clinicians; broad-spectrum: 92.4% vs. 89.3%; pathogen AUROC 0.60–0.68; AMR AUROC 0.59–0.74

a Preprint. ^b^Published conference abstract. Green = Level 1 (deployed); Yellow = Level 2 (clinical evaluation); Orange = Level 3 (retrospective simulation).

The final Table 1 comprised two studies of pneumonia or predominantly pulmonary infection (including the *S. maltophilia* cohort, in which 91% of infections were pulmonary), four of bacteremia/bloodstream infection, two involving urinary tract infection, and two of sepsis. Hospital-acquired infection was not treated as a separate target category.

Three studies used machine learning trained on mass-spectrometry or molecular-diagnostic data to predict resistance or generate treatment recommendations. Several applied gradient-boosted trees, random forests, or logistic regression to electronic health record data. Other approaches included personalized antibiograms with linear programming, a partially observable Markov decision process, and dynamic thresholding to translate pathogen and antimicrobial-resistance probabilities into antibiotic recommendations. ePNa combined real-time EHR data extraction, algorithmic risk stratification, and a conventional guideline engine embedded in clinical workflow.

Sample sizes ranged from 117 blood cultures in a concordance study to 29,508 adults in the Kanjilal retrospective evaluation; the three deployed systems collectively enrolled 8,700 patients in direct clinical use. Designs included randomized and stepped-wedge trials, a before-after study, cross-sectional concordance evaluations, retrospective cohorts, and retrospective model or policy simulations.

### 3.3 Risk of bias

Risk of bias assessments are presented in Table 2. Among the Level 1 studies, the single randomized trial (Lin 2025) was rated as high risk of bias on RoB 2, primarily because outcomes were assessed through unblinded healthcare professional surveys rather than from patient records. The stepped-wedge trial (Dean 2022) (7) was rated as moderate risk on ROBINS-I: confounding was partially addressed by the sequential rollout design, and outcomes were extracted from electronic records. The before-after study (Ho 2024) (18) received a serious risk-of-bias rating on ROBINS-I because it lacked a concurrent control group and was susceptible to temporal confounding and co-interventions.

**Table 2:** Risk-of-bias assessment of included studies.

Level 1 studies: Risk of bias								
Study	D1: Randomization	D2: Deviations	D3: Missing data	D4: Measurement	D5: Reporting	Overall	Direction	
Lin 2025 (17) (RoB 2)	Some concerns (HCP-level cluster randomization)	Some concerns (open-label; HCPs know group)	Low risk (mortality complete)	High risk (HCP surveys, unblinded)	Low risk (pre-registered)	High risk	Favors intervention

Study	D1: Confounding	D2: Selection	D3: Classification	D4: Deviations	D5: Missing data	D6: Measurement	D7: Reporting	Overall
Dean 2022 (7) (ROBINS-I)	Moderate (stepped-wedge adjusts)	Low (all ED pneumonia)	Low (date defined)	Low (pragmatic ITT)	Low (EHR data)	Low (mortality records)	Low (NCT03358342)	Moderate
Ho 2024 (18) (ROBINS-I)	Serious (no concurrent ctrl)	Moderate (time-period)	Low (date clear)	Moderate (co-interventions)	Low (EHR + pharmacy)	Low (records)	Moderate (some NS)	Serious

Level 2–3 studies: PROBAST							
Study	Level	D1: Participants	D2: Predictors	D3: Outcome	D4: Analysis	Overall RoB	Applicability
Gómez de la Torre 2026a (19)	2	Low	Low	Low	Low (concordance; logistic reg; clustering)	Low	High concern (survey-based; single-city Lima; COI)
Gómez de la Torre 2025 (20)	2	Low	Low	Low	Low (prospective; agreement stats)	Low	Moderate (single laboratory; COI)
Feng 2025^a^ (21)	2	Low	Low	Low	Moderate (retro; ext validation but preprint)	Moderate	Moderate (onco-hematology specific)
Lee 2023 (22)	2	Low	Low	Low	Moderate (single-center; no ext validation)	Moderate	High concern (single Korean hospital)
Corbin 2022 (6)	3	Low	Low	Low	Low (multi-site; temporal split; open)	Low	Moderate (US academic centers)
Tsoukalas 2015 (23)	3	Moderate (missing data)	Low	Low	High (POMDP complex; small n; no ext val)	High	High concern (single center; old data)
Kanjilal 2022^b^ (24)	3	Moderate (eligibility and exclusions insufficiently reported)	Moderate (1,200 EHR predictors; measurement and missingness unclear)	Moderate (targets stated; reference-standard and blinding details unclear)	Moderate (temporal split; calibration, missing-data handling, and threshold derivation incompletely reported)	Moderate	Moderate concern (single US health system; limited methodological reporting)

a Preprint. ^b^Published conference abstract. Green = low; Yellow = moderate; Red = high/serious.

Among the seven Level 2 and Level 3 studies assessed with PROBAST, three were classified as low, three as moderate under the descriptive adaptation defined in Methods, and one as high overall risk of bias. Feng 2025 (21) and Lee 2023 (22) were moderate because of analysis-domain concerns. Kanjilal 2022 (24) was classified as moderate because the published conference abstract reported a temporal split and key performance results but provided insufficient information on eligibility, predictor handling, outcome/reference-standard procedures, calibration, missing data, and threshold derivation; no high-risk domain could be established from the available report. Tsoukalas 2015 (23) was high because of a high-risk analysis domain. Applicability concerns were greatest for the Lima OneChoice evaluation and for single-center or highly specific populations. These ratings do not establish clinical effectiveness.

### 3.4 Synthesis of results by implementation level

#### 3.4.1 Level 1: clinically deployed CDSS

Three of 10 studies (30%) reported clinical deployment of AI-based CDSS, meaning that clinicians used the system within the clinical workflow to guide antibiotic prescribing for real patients. These studies differed in infection, design, and geography but constitute the only direct evidence on patient-level effects.

The largest deployed study was a pragmatic stepped-wedge cluster-controlled trial conducted across 16 community hospitals in the United States (Dean 2022) (7).ePNa was an open-loop, real-time CDSS embedded in the electronic health record, extracting patient data to guide pneumonia diagnosis, risk stratification, microbiological workup, site-of-care decisions, and antibiotic therapy. Among 6,848 adult emergency department patients with pneumonia, 30-day all-cause mortality fell after ePNa deployment (adjusted OR 0.62, 95% CI 0.49–0.79, *P* < 0.001), with the reduction consistent across hospital clusters.

Guideline-concordant antibiotic prescribing increased from 83.5% to 90.2% (*P* < 0.001), and the mean time from emergency department arrival to first antibiotic dose decreased from 159 to 151 min (*P* < 0.001). Outpatient disposition from the emergency department rose from 29.2% to 46.9% without a corresponding increase in 7-day secondary hospital admissions (5.2% vs. 6.1%). Clinicians used the tool in 67% of eligible encounters.

The second deployed study was a randomized controlled trial from Taiwan (Lin 2025) (17) that evaluated an AI-CDSS that used MALDI-TOF mass spectrometry data and machine learning to predict antibiotic resistance in *Stenotrophomonas maltophilia* infections, 1 day earlier than conventional susceptibility testing. A total of 400 healthcare professionals managed 1,600 infections, randomized 1:1 to the AI-CDSS or standard practice. The 14-day mortality rate was 11.5% (92/800) in the AI-CDSS group compared with 15.1% (121/800) in the control group (*P* = 0.03). Clinicians in the intervention arm reported significantly higher prescribing confidence (*P* < 0.001) and better antibiotic selection at all survey time points (days 3, 5, 7, and 14). It is also the only study in this review that measured outcomes through clinician surveys, a design prone to reporting bias.

Ho et al. (2024) (18) conducted a before-after study of iAMS, an EHR-integrated AI platform combining sepsis prediction, MRSA detection, a personalized antibiogram, and antibiotic dosing recommendations, in patients with Staphylococcus aureus bacteremia (MRSA/MSSA) at China Medical University Hospital, Taiwan. Among 252 patients (118 iAMS, 134 non-iAMS), MRSA patients had a shorter length of stay after implementation, from 35.6 to 23.3 days (*P* = 0.038), and a higher 45-day discharge rate, up 20.4 percentage points, though this difference did not reach significance (*P* = 0.064). Near-miss medication incidents reported by pharmacists fell by 97.3% (*P* = 0.013). One-year survival was numerically higher with iAMS among MRSA patients (70.9% vs. 53.3%) but did not reach statistical significance (*P* = 0.41).

Taken together, two of the three deployed studies demonstrated statistically significant reductions in mortality, two reported reductions in hospital length of stay or time to antibiotic initiation, and all three showed improvements in antibiotic prescribing adequacy. No harms attributable to CDSS use were reported in the included studies; however, harm ascertainment and false-recommendation consequences were incompletely reported.

#### 3.4.2 Level 2: clinical evaluation without deployment

Four studies (40%) compared CDSS recommendations with physicians' decisions in clinical cases without integrating the system into the treatment workflow. These studies provide evidence of potential decision-support performance, not patient-level clinical effectiveness.

Two studies from Peru (Gomez de la Torre 2026a (19), 2025 (20) evaluated the OneChoice AI-CDSS for the treatment of bacteremia. In the larger study (Gomez de la Torre 2026a) (19), 94 physicians (35 infectious disease specialists and 59 non-specialists) assessed 366 cases of bacteremia. The overall concordance between CDSS and physician decisions was 96.1% when considering any recommended treatment and 74.6% for the top recommendation alone, with substantial inter-rater agreement (kappa = 0.70). Infectious disease specialists showed higher concordance (kappa = 0.78) than non-specialists (kappa = 0.61), and specialization was the strongest predictor of agreement (OR 2.26, *P* = 0.001). The CDSS identified cases where unnecessary carbapenem prescriptions could be avoided. Concordance was highest for *Escherichia coli* and lowest for *Pseudomonas aeruginosa*. In the companion study (Gomez de la Torre 2025) (20), molecular-only CDSS recommendations showed 80.3% concordance with molecular-plus-phenotypic recommendations (kappa = 0.80) and were delivered 29 h earlier, suggesting that AI-CDSS paired with molecular diagnostics can accelerate treatment guidance without a meaningful loss in accuracy.

A multicenter retrospective cohort study from China (Feng 2025) (21) developed machine-learning CDSSs (XGBoost, random forest) for Pseudomonas aeruginosa bloodstream infections in 924 onco-hematology patients across three hospitals. When CDSS recommendations were retrospectively compared with physicians' prescriptions, the rate of inappropriate early antibiotic therapy decreased from 11.9% to 4.0% in one external test set (*P* = 0.01) and from 20.8% to 7.1% in a second external test set (*P* < 0.001). Empirical carbapenem use was also reduced across both test sets. The model showed discrimination for carbapenem-resistant P. aeruginosa across the reported test sets, but these performance estimates did not establish clinical utility. This study was available as a preprint at the time of our search and was therefore interpreted as preliminary evidence.

A qualitative sensitivity appraisal excluding Feng 2025 (21)did not materially alter the interpretation of the Level 2 evidence. The remaining studies continued to suggest potential decision-support utility but provided no evidence of patient-level clinical effectiveness because none involved prospective clinical deployment. Excluding Feng further reduced the breadth of the Level 2 evidence and the available evidence for multicenter external validation. A single-center retrospective study from South Korea (Lee 2023) (22) developed a gradient-boosted decision tree model to predict ciprofloxacin resistance and the presence of extended-spectrum beta-lactamases (ESBLs) in 550 emergency department patients with urinary tract infections, using 39 clinical variables. When the model's recommendations were compared with actual emergency department prescriptions, the probability of selecting an ineffective antibiotic was reduced by 20%. The model maintained performance when the predictor set was narrowed from 39 to as few as five variables, suggesting feasibility for point-of-care deployment.

Across the four Level 2 studies, the common thread was concordance between AI-CDSS recommendations and expert decisions, with evidence that the systems could reduce unnecessary use of broad-spectrum antibiotics. However, calibration, clinically justified thresholds, external or temporal validation, clinical net benefit, workflow feasibility, and the consequences of false recommendations were incompletely reported. None of the four systems was used to guide prescribing in a real clinical setting, so translation of any apparent benefit to patients remains unproven.

#### 3.4.3 Level 3: retrospective simulation

Three studies (30%) evaluated AI-CDSS recommendations or policies retrospectively on historical patient data without prospective deployment: Corbin 2022 (6), Tsoukalas 2015 (23), and Kanjilal 2022 (24).

A multi-site study from the United States (Corbin 2022) (6) developed personalized antibiograms using machine learning to optimize empiric antibiotic selection across two large datasets: 8,342 emergency department infections from Stanford and 15,806 uncomplicated UTIs from Boston. The personalized antibiograms achieved an antibiotic coverage rate of 85.9% at Stanford (vs. 84.3% for clinicians, *P* = 0.11) and 90.4% in Boston (vs. 88.1% for clinicians, *P* < 0.0001). The greater contribution, however, was spectrum narrowing: 69% of empiric vancomycin plus piperacillin-tazobactam prescriptions at Stanford were narrowed to piperacillin-tazobactam alone while maintaining equivalent coverage, and 48% of ciprofloxacin prescriptions in Boston were narrowed to trimethoprim-sulfamethoxazole.

The oldest study in this review (Tsoukalas 2015) (23) used a partially observable Markov decision process (POMDP) to derive optimal antibiotic administration policies for 1,492 sepsis patients from a single US center. Data-derived antibiotic policies led to favorable patient outcomes in 49% of simulated cases, compared with 37% under non-policy trajectories (*P* = 1.3 × 10^−13^). The model predicted mortality with an AUC of 0.70 and length of stay with AUCs ranging from 0.69 to 0.73.

Kanjilal et al. (2022) (24), available as a published conference abstract, retrospectively evaluated 29,508 adults with a community-onset Adult Sepsis Event in the Mass General Brigham health system. Logistic regression, random forest, and XGBoost models predicted pathogens and antimicrobial resistance; dynamic thresholding converted probabilities into phenotypes and selected the narrowest antibiotic predicted to be susceptible. Compared with clinicians' decisions, the model reduced inappropriately narrow treatment from 9.4% (95% CI 9.1%–9.6%) to 7.3% (95% CI 7.1%–7.6%) but increased broad-spectrum treatment from 89.3% (95% CI 88.9%–89.6%) to 92.4% (95% CI 92.2%–92.7%). Pathogen-prediction AUROCs ranged from 0.60 to 0.68 and antimicrobial-resistance AUROCs from 0.59 to 0.74. These retrospective results show a trade-off between undertreatment and spectrum reduction and do not demonstrate patient-level effectiveness or clinical deployment.

All three Level 3 studies provided computational or retrospective evidence only. None prospectively deployed the system or used its recommendations to guide actual prescribing; accordingly, their performance estimates and simulated prescribing changes should not be interpreted as demonstrated clinical effectiveness. Kanjilal was available only as a published conference abstract with limited methodological reporting.

### 3.5 The implementation gap

Of 10 eligible AI-CDSS studies, three (30%) were clinically deployed and seven (70%) remained at the evaluation or simulation stage. The non-deployed evidence included recent publications as well as the 2015 POMDP study, so recency alone did not explain the gap. Several systems were developed within a single institution or localized health system; Kanjilal reported data from one US health system but did not specify the number of contributing sites in the published conference abstract. The deployed systems shared practical enablers such as EHR integration or use of established laboratory infrastructure, whereas most non-deployed studies relied on retrospective data and did not describe the operational requirements for real-time use.

### 3.6 Certainty of evidence

Certainty of evidence was low for mortality/survival and very low for the remaining clinically relevant outcomes (Supplementary Table S4).

## 4 Discussion

### 4.1 Principal finding

The principal finding is not simply that AI-based CDSS may improve antibiotic prescribing, but that most evaluated systems have not progressed to clinical implementation. Only three of 10 studies were deployed, while seven remained at clinical evaluation or retrospective simulation. This 30% deployment proportion is consistent with broader concerns that many validated healthcare AI models do not reach clinical workflows (10, 18, 25). Computational performance and retrospective concordance cannot substitute for evidence that clinicians can use a system safely and effectively in real time.

### 4.2 Why does this implementation gap exist?

The discrepancy between the high diagnostic performance of AI models and their limited implementation in clinical practice is driven by several intertwined barriers. First, many clinical decision support systems (CDSS) necessitate manual data entry or function independently of standard clinical workflows, thereby imposing a significant additional workload on healthcare providers. This contributes to low adoption rates, even when they demonstrate high predictive performance (26, 27). Corroborating this, our review demonstrated that the systems achieving the highest deployment benchmarks (ePNa, Lin 2025's AI-CDSS, and iAMS) featured native integration within electronic health records. Conversely, none of the remaining models evaluated reported this level of systemic interoperability.

Second, the regulatory and institutional processes required to incorporate AI tools into clinical practice are often lengthy and cumbersome. High performance is only the first step before adoption, as institutional evaluations and reviews related to cybersecurity, usage time, staff training, and other factors are necessary, which can significantly impact implementation (28). Furthermore, we must consider the acceptance of these systems by healthcare professionals. Although Lin et al. (2025) observed that physicians reported greater confidence in prescribing medication with the support of an AI-based clinical decision support system (CDSS), other studies show that there is still reluctance to follow algorithmic recommendations, especially when these are perceived as a replacement for clinical judgment (26, 29).

External validation was limited. All seven non-deployed systems were evaluated within a single country; Corbin 2022 (6) and Feng 2025 (21) included multiple domestic sites, while several others were confined to one institution or a localized diagnostic context. Kanjilal used one US health system, but the published conference abstract did not report the number of sites. Local microbiology, formularies, resistance patterns, and workflows vary across hospitals; therefore, models require independent external or temporal validation, local calibration, and decision-utility assessment before deployment.

Maintaining models after implementation presents an additional challenge. Bacterial resistance patterns, formularies, diagnostic technologies, and clinical workflows evolve over time, creating risks of temporal data drift and declining calibration. Safe deployment therefore requires predefined performance monitoring, periodic recalibration or retraining when justified, surveillance for false recommendations and unintended prescribing effects, and reassessment of transportability across hospitals and populations. None of the non-deployed studies described a post-deployment updating or monitoring strategy (30).

Finally, legal responsibility for the use of these tools is still not clearly defined (28). It is necessary to answer the question: who assumes responsibility when a recommendation generated by an AI system contributes to an adverse event? Furthermore, CDSS that recommend a specific antibiotic, its dosage, or the duration of treatment are usually classified as medical software and are therefore subject to increasingly stringent regulatory frameworks, such as those established by the FDA (25) and by European legislation, which includes the Medical Devices Regulation (31) and the Artificial Intelligence Act (32). The latter considers that most clinical AI applications correspond to high-risk systems and requires specific data governance requirements, human oversight, and post-market surveillance. None of the included studies reported having obtained regulatory authorization. This finding reinforces that safe integration into clinical practice requires more than high-performing algorithms; it also requires governance, human oversight, validation in the intended setting, monitoring, and clearly defined accountability.

Generative language models represent the most recent addition to the field of decision support in antimicrobial therapy; however, they introduce particular risks, such as the generation of plausible but incorrect responses (“hallucinations”) and a high sensitivity to the way queries are phrased.

None of the included studies evaluated this type of model; however, their eventual implementation will likely require additional safety mechanisms related to how they retrieve evidence, clinician oversight, and assessments focused on patient safety. Taken together, these challenges reinforce the idea that the main challenge for AI in optimizing antibiotic therapy lies not in developing more sophisticated algorithms, but in ensuring that they can be safely, reliably, and sustainably integrated into clinical practice.

### 4.3 What do the deployed studies show?

Despite the small number of deployed systems, the three Level 1 studies provide preliminary signals of benefit that should be interpreted cautiously. Two of three reported statistically significant mortality reductions: Dean 2022 (7) demonstrated a 38% reduction in 30-day mortality for pneumonia after ePNa deployment (aOR 0.62), and Lin 2025 showed a 3.6-percentage-point reduction in 14-day mortality for S. maltophilia infections (11.5% vs. 15.1%). Hospital processes also improved: time to first antibiotic was reduced in the ePNa trial, length of stay decreased in the iAMS study, and guideline concordance increased in both Dean 2022 (83.5% to 90.2%) and Lin 2025. Medication safety also improved, with a 97.3% reduction in near-miss medication incidents after iAMS deployment (Ho 2024) (18).

These findings should be interpreted with caution. The three deployed studies evaluated different CDSS platforms, targeted different infections in different countries, and employed different study designs (one stepped-wedge trial, one parallel-group RCT with survey-based outcomes, and one uncontrolled before-after study). Their risk of bias ranged from moderate (stepped-wedge trial) to serious (before-after study, owing to unmeasured confounding), and the RCT relied on healthcare professionals' self-reports for key outcomes. The most rigorously conducted example—a stepped-wedge trial that adjusted for baseline illness severity and used sensitivity analyses to exclude secular trends—illustrates the design standard to which future deployment studies should aspire. Quantitative pooling would not be appropriate. Within individual studies, deployment was associated with improvements across multiple clinically relevant outcomes, and no harms were reported; however, incomplete harm ascertainment, heterogeneity, and the observational or unblinded nature of most designs support a low-certainty interpretation rather than a causal conclusion.

### 4.4 Implications for research and practice

The evidence suggests promising technological development but incomplete clinical readiness. The seven non-deployed studies reported concordance, simulated prescribing changes, or predictive performance, but these findings did not establish patient benefit. Progression toward prospective deployment should require adequate calibration, clinically justified thresholds, external or temporal validation, clinical net benefit, and explicit assessment of false-recommendation consequences (33).

Specifically, future studies should prioritize: (a) pragmatic, multicenter trials that evaluate patient outcomes rather than model performance metrics alone; (b) effectiveness-implementation hybrid designs (34, 35) that simultaneously measure clinical effectiveness and implementation determinants; (c) head-to-head comparisons of AI-CDSS against conventional antimicrobial stewardship programs (36); and (d) economic evaluations of development, integration, and maintenance costs. Particular attention is needed in low- and middle-income settings, where antimicrobial-resistance burdens are high and diagnostic infrastructure is constrained. Two of the 10 studies were conducted in Peru, showing that CDSS can be developed and evaluated outside high-income health systems.

Our findings both extend and reframe the existing literature. Previous comparative evidence has suggested that machine-learning approaches may improve selected performance measures relative to conventional risk scores in some antimicrobial-stewardship settings, while also showing heterogeneity and limited external validation (11). Recent infectious-disease AI reviews have similarly emphasized that discrimination alone does not establish clinical utility and that evidence maturity depends on validation, calibration, and implementation context (13, 33). These observations strengthen the rationale for focusing this review on clinical deployment and patient-relevant outcomes rather than on predictive performance alone. Prior syntheses of AI in infectious disease have catalogued applications across tuberculosis, sepsis, and antimicrobial stewardship and have appraised model performance and, more recently, evidence maturity (8, 9, 37, 38). By restricting the scope to antibiotic-therapy CDSS in hospitalized adults and by classifying studies according to their level of clinical implementation, we shift the evaluative lens from how well models perform in silico to whether they have reached—and benefited—patients. This deployment-oriented framing helps explain an apparent paradox in the field: reviews consistently report strong algorithmic performance, yet real-world impact remains scarce because, so few systems are ever deployed (39). Clinical deployment should therefore complement, rather than replace, established antimicrobial stewardship structures and practices (40). Positioning individual studies along an implementation continuum, rather than pooling performance metrics, offers a more faithful picture of translational readiness.

The two OneChoice evaluations warrant particular caution. Both were conducted within a limited Lima-based diagnostic context and assessed concordance or recommendation timing rather than prospective clinical deployment. Several review authors were also authors of these studies, and relevant commercial relationships are disclosed in the Conflict of Interest statement. Although screening, extraction, and appraisal were conducted independently according to the stated firewall, these relationships do not eliminate residual concerns regarding sponsorship bias, self-evaluation, applicability, and transportability. Consistent with Table 2, applicability was rated as high concern for Gómez de la Torre et al. (2026a) (19), given its survey-based single-city setting and conflict-of-interest considerations, and moderate concern for Gómez de la Torre et al. (2025), which was conducted in a single laboratory setting. These findings should therefore not be interpreted as evidence of patient-level effectiveness or broad generalizability, and independent multicenter prospective evaluations remain necessary.

### 4.5 Strengths and limitations

This systematic review has several strengths. To our knowledge, it is the first review to classify AI-CDSS studies for antibiotic therapy by their level of clinical implementation, moving beyond the typical focus on model performance to address the translational gap between development and deployment. The three-level classification (deployed, clinically evaluated, retrospectively simulated) allowed us to separate evidence of actual clinical effectiveness from evidence of computational promise. We used design-appropriate risk-of-bias tools (RoB 2, ROBINS-I, PROBAST) rather than applying a single instrument across heterogeneous study types, and we searched four electronic databases without date or language restrictions.

This framework should not be interpreted as a formal technology-readiness scale. It is a pragmatic classification of the clinical implementation evidence available in the included studies.

This review has several limitations. The small evidence base (*n* = 10) restricts inference. PubMed/MEDLINE, Web of Science, and Scopus used mandatory hard-clinical-outcome terms, whereas Embase did not; this asymmetry may have under-identified studies focused on prescribing appropriateness, time to therapy, spectrum narrowing, guideline adherence, or model performance, particularly at Levels 2 and 3. Clinical and methodological heterogeneity precluded meta-analysis. The scope was restricted to pneumonia, urinary tract infection, bacteremia/bloodstream infection, and sepsis. Six studies were confined to a single institution or narrowly defined single-city setting, while the number of sites contributing to Kanjilal was not reported. Publication bias cannot be excluded, and trial registries, grey-literature databases, and preprint servers were not searched. Feng 2025 remained a preprint; excluding it did not materially change the Level 2 interpretation but reduced the breadth of multicenter evidence. Kanjilal was available only as a published conference abstract with limited methodological reporting, which constrained risk-of-bias appraisal and precluded verification of unreported methods. Formal outcome-specific GRADE was introduced during peer review; certainty was low for mortality/survival and very low for the remaining clinically relevant outcomes (Supplementary Table S4). New studies may have appeared after the June 28, 2026 search.

## 5 Conclusions

This systematic review identified a pronounced implementation gap: three of 10 eligible studies (30%) deployed an AI-based CDSS in clinical practice, whereas seven (70%) remained at clinical evaluation or retrospective simulation. Deployed systems showed favorable signals for mortality, hospital processes, prescribing adequacy, and medication safety, but these findings derive from a small, heterogeneous evidence base of low to very-low certainty, depending on the outcome. Non-deployed systems, including the Kanjilal published conference abstract, demonstrated computational or retrospective performance rather than clinical effectiveness. Closing the gap requires prospective multicenter deployment studies, external validation and calibration, safe workflow integration, monitoring for false recommendations and data drift, and sustained implementation infrastructure.

## Data Availability

The original contributions presented in the study are included in the article/Supplementary Material, further inquiries can be directed to the corresponding author.

## Supplementary material

Supplementary Material S1 Complete search strategies for PubMed, Scopus, Web of Science, and Embase

Supplementary Material S2 Full-text selection decisions and data-extraction information for the 10 included studies.

Supplementary Material S3 Eligibility criteria applied during screening and full-text assessment

Supplementary Material S4 GRADE certainty-of-evidence assessment for clinically deployed AI-based CDSS (Level 1).
