# Supplementary material for: AI-based clinical decision support systems for antibiotic therapy in common adult infections: a systematic review from development to clinical implementation
Source: Front Digit Health. 2026 Sep 30;8:1944745. doi: 10.3389/fdgth.2026.1944745 (PMC13662083; doi:10.3389/fdgth.2026.1944745)
Supplement: Supplementary file 1 [file Datasheet1.docx]

**Supplementary Material**

*AI-Based Clinical Decision Support Systems for Antibiotic Therapy in Common Adult Infections: A Systematic Review from Development to Clinical Implementation*

**Supplementary Table S1. Search strategies**

Search-strategy development began on June 7, 2026. The definitive searches were executed on June 28, 2026, after submission of the PROSPERO record. No date or language restrictions were applied.

| **Database** | **Search strategy** | **Results** |
| --- | --- | --- |
| PubMed/MEDLINE | ("clinical decision support systems"[Mesh] OR "clinical decision support" OR CDSS OR "decision support tool" OR "decision support software") AND (artificial intelligence OR machine learning OR deep learning OR AI) AND (antibiotic* OR antimicrobial* OR culture* OR bacteremia OR pneumonia OR "urinary tract infection" OR UTI) AND (mortality OR death OR survival OR "length of stay" OR hospitalization OR "hospital stay") | 302 |
| Web of Science | TS=(("clinical decision support" OR CDSS OR "decision support tool" OR "decision support software" OR "clinical decision support system*") AND ("artificial intelligence" OR "machine learning" OR "deep learning" OR AI) AND (antibiotic* OR antimicrobial* OR culture* OR bacteremia OR pneumonia OR "urinary tract infection" OR UTI) AND (mortality OR death OR survival OR "length of stay" OR hospitalization OR "hospital stay")) | 97 |
| Scopus | TITLE-ABS-KEY(("clinical decision support" OR CDSS OR "decision support tool" OR "decision support software" OR "clinical decision support system*") AND ("artificial intelligence" OR "machine learning" OR "deep learning" OR AI) AND (antibiotic* OR antimicrobial* OR culture* OR bacteremia OR pneumonia OR "urinary tract infection" OR UTI) AND (mortality OR death OR survival OR "length of stay" OR hospitalization OR "hospital stay")) | 172 |
| Embase | ('clinical decision support system'/exp OR 'clinical decision support':ti,ab,kw OR cdss:ti,ab,kw OR 'decision support tool':ti,ab,kw OR 'decision support software':ti,ab,kw) AND ('artificial intelligence'/exp OR 'machine learning'/exp OR 'deep learning'/exp OR 'artificial intelligence':ti,ab,kw OR 'machine learning':ti,ab,kw OR 'deep learning':ti,ab,kw OR ai:ti,ab,kw) AND ('antiinfective agent'/exp OR antibiotic*:ti,ab,kw OR antimicrobial*:ti,ab,kw OR culture*:ti,ab,kw OR bacteremia:ti,ab,kw OR pneumonia:ti,ab,kw OR 'urinary tract infection':ti,ab,kw OR uti:ti,ab,kw) | 586 |
| Total |  | 1157 |

Note: Additional records were identified by hand-searching reference lists of included studies and relevant reviews. Authors or experts were not contacted. CENTRAL was not searched.

**Supplementary Table S2. Full-text selection: consensus decisions for the 13 studies assessed**

After independent screening, 13 unique reports were assessed for eligibility. Ten were included and three were excluded. The table below presents the consensus decision and rationale for each report.

| **First author** | **Year** | **Title** | **Decision** | **Rationale** |
| --- | --- | --- | --- | --- |
| Dean et al. | 2022 | A pragmatic, stepped-wedge, cluster-controlled clinical trial of real-time pneumonia clinical decision support | INCLUDE | Level 1. CDSS deployed in 16 hospitals. Pneumonia (n=6,848). Hard clinical outcomes: mortality OR 0.62 (P<0.001), time to Abx reduced, concordance improved. |
| Lin et al. | 2025 | Implementing an AI-enhanced CDSS for Stenotrophomonas maltophilia: a survey-based RCT | INCLUDE | Level 1. Only RCT. CDSS deployed vs standard care (n=1,600). Mortality reduced (11.5% vs 15.1%, P=0.03). Caveat: organism-specific, but 91% pulmonary infections. |
| Ho et al. | 2024 | Impact of iAMS on clinical outcomes among patients with bacteraemia caused by MRSA | INCLUDE | Level 1. Before-after implementation. CDSS deployed hospital-wide (n=252). LOS reduced (P=0.038); medication incidents reduced 97.3% (P=0.013). |
| Gómez de la Torre et al. | 2026 | Comparison of OneChoice AI-based CDSS recommendations with ID specialists and non-specialists for bacteremia (Lima) | INCLUDE | Level 2. CDSS recommendations compared with 94 physicians for 366 bacteremia cases. Concordance κ=0.70. Reduced inappropriate carbapenem prescribing. |
| Gómez de la Torre et al. | 2025 | AI-based treatment recommendations enhance speed and accuracy in bacteremia management | INCLUDE | Level 2. Prospective evaluation comparing molecular-only vs molecular+phenotypic CDSS recommendations (n=117). Concordance 80.3%; molecular recs 29h earlier. |
| Feng et al. | 2025 | ML-guided CDSS for early antibiotic selection in P. aeruginosa BSI among onco-hematology patients | INCLUDE | Level 2. Multicenter (n=924). CDSS vs physician decisions. Inappropriate therapy reduced from 11.9% to 4.0% (P=0.01). Caveat: preprint; specific population. |
| Lee et al. | 2023 | Machine learning model for predicting ciprofloxacin resistance and presence of ESBL in patients with UTI in the ED | INCLUDE | Level 2. ML model compared with actual ED prescribers for UTI (n=550). Ineffective antibiotic use reduced 20%. Single-center retrospective. |
| Corbin et al. | 2022 | Personalized antibiograms for machine learning driven antibiotic selection | INCLUDE | Level 3. Retrospective multi-site simulation (n=24,148). ML vs clinician prescribing. Coverage 90.4% vs 88.1% (P<0.0001); narrowed broad-spectrum antibiotics. |
| Tsoukalas et al. | 2015 | From data to optimal decision making: a data-driven probabilistic ML approach to decision support for patients with sepsis | INCLUDE | Level 3. POMDP model for antibiotic policies in sepsis (n=1,492). Policy-concordant treatment: 49% favorable vs 37% (P=1.3e-13). Retrospective simulation. |
| Kanjilal et al. | 2022 | Antimicrobial stewardship for empirical treatment of bloodstream infection using ML clinical decision support | INCLUDE | Level 3. Published conference abstract. Retrospective model evaluation in 29,508 adults with community-onset sepsis; model-generated antibiotic recommendations were compared with clinicians' decisions. The model reduced inappropriately narrow treatment (7.3% vs 9.4%) but increased broad-spectrum use (92.4% vs 89.3%). No prospective clinical deployment. |
| Wackerbarth et al. | 2025 | Electronic decision support for pneumonia is associated with reduced outcome disparities | EXCLUDE | Secondary analysis of the same cohort reported in Dean 2022 (same 6,848 patients, same 16 hospitals, same ePNa intervention). Excluded to avoid double-counting. |
| Williams et al. | NR | Antibiotic clinical decision support for pneumonia in the ED: a randomized trial | EXCLUDE | Confirmed pediatric population after full-text review (author: Derek J. Williams, Vanderbilt pediatric infectious diseases). Does not meet population criterion. |
| Düvel et al. | NR | An AI-based CDSS for antibiotic therapy in sepsis (KINBIOTICS): use case analysis | EXCLUDE | System acceptability and usability study without clinical outcomes. The study describes the KINBIOTICS system architecture and HCP acceptance; no comparative clinical data. |

NR, not reported. Included in final synthesis: n = 10. Excluded after eligibility assessment: n = 3. Level 1 = deployed in clinical practice; Level 2 = evaluated against clinical practice without deployment; Level 3 = retrospective simulation/evaluation. Kanjilal et al. was available as a published conference abstract.

**Supplementary Table S3. Eligibility criteria applied during screening and full-text assessment**

| **Criterion** | **Inclusion** | **Exclusion** |
| --- | --- | --- |
| Population | Adults (>=18 years) hospitalized with pneumonia, urinary tract infection, bacteremia/bloodstream infection, or sepsis. Hospital-acquired infections were eligible only when occurring within one of these syndromes. | Pediatric populations; outpatient settings; non-infectious conditions; healthy volunteers |
| Intervention | Computerized systems using data-driven learning, probabilistic inference, or data-driven optimization to generate or modify individualized antibiotic-treatment recommendations. | Purely deterministic guideline engines; conventional risk scores without a data-driven component; manual stewardship without algorithmic decision support; diagnostic-only AI without treatment recommendations. |
| Comparator | Standard clinical practice, physician prescribing decisions (prospective or retrospective), or pre-implementation periods | No comparator at all; studies comparing two AI systems without reference to clinical practice |
| Outcomes | Mortality; length of stay; time to appropriate antibiotic; antibiotic concordance/appropriateness; coverage rates; spectrum narrowing; adverse drug events; antibiotic costs | Only model performance metrics without any comparison to clinical practice (e.g., AUC alone without clinical context) |
| Study design | Primary studies: RCTs, quasi-experimental, cohort, cross-sectional, retrospective model evaluations with clinical comparison | Systematic reviews; narrative reviews; editorials; letters; protocols; qualitative studies; case reports; instrument validation without clinical comparison |

**Supplementary Table S4. GRADE certainty of evidence for clinically deployed AI-based CDSS (Level 1)**

**Scope:** Outcome-specific certainty assessment of patient-level and clinically relevant process outcomes from the three Level 1 studies. Level 2 and Level 3 studies were not used to infer patient-level clinical effectiveness because their systems were not prospectively deployed in clinical care.

| **Outcome** | **Contributing evidence** | **Main finding(s)** | **Risk of bias** | **Inconsistency** | **Indirectness** | **Imprecision** | **Publication bias** | **Overall certainty** |
| --- | --- | --- | --- | --- | --- | --- | --- | --- |
| Mortality / survival | Dean 2022 (stepped-wedge cluster-controlled; n=6,848); Lin 2025 (RCT; n=1,600); Ho 2024 (before-after; n=252) | Dean: 30-day mortality aOR 0.62 (95% CI 0.49-0.79). Lin: 14-day mortality 11.5% vs 15.1% (P=0.03). Ho: 1-year survival in MRSA 70.9% vs 53.3% (P=0.41). | Serious - moderate/high/serious risk-of-bias concerns across the three studies, including residual confounding in non-randomized designs and limitations of the randomized study. | Not serious - all estimates were directionally favorable, although one was not statistically significant and effect magnitudes/time horizons differed. | Serious - different infections, CDSS platforms, settings, and mortality time points limit inference to a general class effect of AI-CDSS. | Not serious overall - the two largest studies provided reasonably informative mortality data; Ho was imprecise but contributed little weight to the overall interpretation. | Not detected; cannot be excluded because only three deployed studies were available. | **LOW** |
| Antibiotic appropriateness / concordance | Dean 2022; Lin 2025; Ho 2024 | Dean: guideline concordance 83.5% to 90.2% (P<0.001). Lin: improved antibiotic selection at all survey time points (P<0.001). Ho: prescribing/safety recommendations improved after iAMS implementation. | Serious - Lin relied substantially on unblinded healthcare-professional assessments; Dean had residual confounding; Ho lacked a concurrent control. | Not serious - findings were consistently favorable, although measures were not directly comparable. | Serious - heterogeneous definitions of appropriateness/concordance and different infections, workflows, and CDSS platforms. | Serious - confidence intervals were incompletely reported for several process measures and no pooled estimate was possible. | Not detected; cannot be excluded. | **VERY LOW** |
| Time to first / appropriate antibiotic therapy | Dean 2022 | Mean time to first antibiotic decreased from 159 to 151 minutes (P<0.001). | Serious - non-randomized stepped-wedge evidence with residual confounding despite adjustment. | Not applicable - single study. | Not serious - directly measured in the intended clinical workflow, although evidence comes from one health system. | Serious - no confidence interval for the time difference was reported in the review and the clinical importance of an ~8-minute reduction is uncertain. | Not detected; cannot be assessed from a single study. | **VERY LOW** |
| Hospital length of stay / discharge | Ho 2024 | Among MRSA patients, length of stay decreased from 35.6 to 23.3 days (P=0.038); 45-day discharge increased by 20.4 percentage points but was not statistically significant (P=0.064). | Serious - uncontrolled before-after design, temporal confounding, and possible co-interventions. | Not applicable - single study. | Serious - single-center MRSA/MSSA bacteremia population and one proprietary implementation context. | Serious - small sample and one non-significant discharge outcome. | Not detected; cannot be assessed from a single study. | **VERY LOW** |
| Medication safety / near-miss incidents | Ho 2024 | Near-miss medication incidents reported by pharmacists decreased by 97.3% (P=0.013). | Serious - uncontrolled before-after design and susceptibility to temporal changes/co-interventions. | Not applicable - single study. | Serious - near-miss incidents are a process/safety surrogate and were assessed in a single institution. | Serious - event counts and confidence intervals were not reported in the review, limiting precision assessment. | Not detected; cannot be assessed from a single study. | **VERY LOW** |
| CDSS-attributable harms / adverse drug events | All three Level 1 studies | No harms attributable to CDSS use were reported; however, harm ascertainment and consequences of false recommendations were incompletely reported. | Serious - incomplete and heterogeneous ascertainment of harms. | Not serious - no contradictory harm signal was reported, but absence of events cannot be interpreted as evidence of safety. | Serious - harms were not consistently defined or actively monitored across studies. | Serious - sparse/absent event data do not exclude clinically important harms. | Not detected; cannot be excluded. | **VERY LOW** |

**GRADE interpretation.** High certainty: very confident that the true effect lies close to the estimate; Moderate: moderately confident; Low: limited confidence; Very low: very little confidence in the effect estimate. Certainty was assessed by outcome using the GRADE domains of risk of bias, inconsistency, indirectness, imprecision, and publication bias. Because quantitative pooling was not appropriate, effect estimates are presented narratively. Judgments concern the body of clinically deployed evidence and should not be inferred from implementation level alone.

**Abbreviations:** AI, artificial intelligence; CDSS, clinical decision support system; CI, confidence interval; aOR, adjusted odds ratio; MRSA, methicillin-resistant Staphylococcus aureus; RCT, randomized controlled trial.

**Important methodological note:** This table grades certainty for clinical effectiveness/safety outcomes from Level 1 studies. Level 2 and Level 3 studies may inform technological performance and readiness, but they do not provide direct evidence that deployment improves patient outcomes.
